# Branched-Chain Amino Acid Database Integrated in MEDIPAD Software as a Tool for Nutritional Investigation of Mediterranean Populations

**DOI:** 10.3390/nu10101392

**Published:** 2018-10-01

**Authors:** Sara Haydar, Thomas Paillot, Christophe Fagot, Yannick Cogne, Athanasios Fountas, Yildiz Tutuncu, Madalina Vintila, Agathocles Tsatsoulis, Pham Thanh Chi, Patrick Garandeau, Dan Chetea, Corin Badiu, Monica Gheorghiu, Dorina Ylli, Corinne Lautier, Morana Jarec, Louis Monnier, Christophe Normand, Jelena Šarac, Abdelhamid Barakat, Sasa Missoni, Michel Pugeat, Patrick Poucheret, Felicia Hanzu, Ramon Gomis, Josep Maria Macias, Serghey Litvinov, Elza Khusnutdinova, Catalina Poiana, Renato Pasquali, Davide Lauro, Giorgio Sesti, Vincenzo Trischitta, Sonia Abdelhak, Akila Zenati, Agron Ylli, Ilhan Satman, Timo Kanninen, Yves Rinato, Florin Grigorescu

**Affiliations:** 1 Unité Mixte de Recherche (UMR)204 NUTRIPASS (Nutrition et Alimentation des Populations aux Suds, IRD, UM, SupAgro), Molecular Endocrinology, Institut Universitaire de Recherche Clinique (IURC), Faculty of Medicine, University of Montpellier, 34093 Montpellier, France; sara.haydar@inserm.fr (S.H.); yannick.cogne@hotmail.fr (Y.C.); phamtc1@gmail.com (P.T.C.); patrick.garandeau@gmail.com (P.G.); corinne.lautier@umontpellier.fr (C.L.); louis.monnier@inserm.fr (L.M.); christophe.normand@inserm.fr (C.N.); 2 Intactile Design SA, 34000 Montpellier, France; paillot.t@intactile.com (T.P.); fagot.c@intactile.com (C.F.); rinato.y@intactile.com (Y.R.); 3 Department of Endocrinology, School of Medicine, University of Ioannina, 45110 Ioannina, Greece; thanasisfountas@gmail.com (A.F.); atsatsou@uoi.gr (A.T.); 4 Department of Internal Medicine, Istanbul University, 34093 Istanbul, Turkey; tutuncuy@yahoo.com (Y.T.); satmandiabet@gmail.com (I.S.); 5 Department of Endocrinology, Universitatea de Medicina si Farmacie Carol Davila, 011863 Bucharest, Romania; madalina.vintila@yahoo.com (M.V.); badicrin@yahoo.co.uk (C.B.); monicagheorghiu@yahoo.com (M.G.); endoparhon@gmail.com (C.P.); 6 Nicolae Paulescu National Institute, 020475 Bucharest, Romania; chetadan@gmail.com; 7 Faculty of Medicine, Mjekesise University of Tirana, 1005 Tirana, Albania; doriylli@yahoo.com (D.Y.); ylliagron@yahoo.com (A.Y.); 8 Institute for Anthropological Research, 10000 Zagreb, Croatia; morana.jarec@inantro.hr (M.J.); jsarac@inantro.hr (J.Š.); sasa.missoni@inantro.hr (S.M.); 9 Institut Pasteur du Maroc, 20360 Casablanca, Morocco; barakat.abdelhamid@yahoo.fr; 10 Fédération d’Endocrinologie, Cardio-Neuro Hospital, University Claude Bernard de Lyon 1, 69677 Lyon-Bron, France; michel.pugeat@chu-lyon.fr; 11 Faculty of Pharmacy, UMR 95 Qualisud, University of Montpellier, 34398 Montpellier, France; patrick.poucheret@umontpellier.fr; 12 Institut d’Investigacions Biomediques August Pi i Sunyer, 08036 Barcelona, Spain; afhanzu@yahoo.com (F.H.); ramon.gomis@idibaps.org (R.G.); 13 Institut Catala d’Arqueologia Classica, 43003 Tarragona, Spain; jmmacias@icac.cat; 14 Institut Biohimii i Genetiki RAN, 450054 Ufa, Russian; seregtg@gmail.com (S.L.); elzakh@rambler.ru (E.K.); 15 Division of Endocrinology, University Alma Mater Studiorum, 40138 Bologna, Italy; renato.pasquali@unibo.it; 16 Department of Internal Medicine, Universita degli Studi di Roma Tor Vergata, 00173 Roma, Italy; d.lauro@med.uniroma2.it; 17 Department of Experimental and Clinical Medicine, University Magna Graecia of Catanzaro, 88100 Catanzaro, Italy; sesti@unicz.it; 18 Scientific Institute Casa Sollievo della Sofferenza, 71013 San Giovanni Rotondo, Italy; vincenzo.trischitta@uniroma1.it; 19 Institut Pasteur de Tunis, Laboratory of Biomedical Genomics and Oncogenetics, 1002 Tunis, Tunisia; sonia.abdelhak@pasteur.rns.tn; 20 Laboratoire de Biochimie Génétique, CHU Bab-El-Oued, Université d’Alger, Alger 16000, Algeria; akilaze@gmail.com; 21 BC Platforms Ltd. Oy, 02130 Espoo, Finland; timo.kanninen@bcplatforms.com

**Keywords:** BCAA, 24-h dietary recall, computer technology, nutrition, dietary assessment, insulin resistance, Mediterranean, web-based recalls, metabolic syndrome

## Abstract

Branched-chained amino acids (BCAA) are essential dietary components for humans and can act as potential biomarkers for diabetes development. To efficiently estimate dietary intake, we developed a BCAA database for 1331 food items found in the French Centre d’Information sur la Qualité des Aliments (CIQUAL) food table by compiling BCAA content from international tables, published measurements, or by food similarity as well as by calculating 267 items from Greek, Turkish, Romanian, and Moroccan mixed dishes. The database embedded in MEDIPAD software capable of registering 24 h of dietary recalls (24HDR) with clinical and genetic data was evaluated based on archived 24HDR of the Saint Pierre Institute (France) from 2957 subjects, which indicated a BCAA content up to 4.2 g/100 g of food and differences among normal weight and obese subjects across BCAA quartiles. We also evaluated the database of 119 interviews of Romanians, Turkish and Albanians in Greece (27–65 years) during the MEDIGENE program, which indicated mean BCAA intake of 13.84 and 12.91 g/day in males and females, respectively, comparable to other studies. The MEDIPAD is user-friendly, multilingual, and secure software and with the BCAA database is suitable for conducting nutritional assessment in the Mediterranean area with particular facilities for food administration.

## 1. Introduction

Obesity, type 2 diabetes (T2D), and metabolic syndrome (MetS) are complex disorders associated with insulin resistance, and because of their long-term metabolic and cardiovascular complications, they can have a severe impact on human morbidity and mortality [1]. During their pathogenesis, genetic susceptibility interacts with environmental factors such as dietary macro- or micronutrients and accurate assessment of dietary intake is an essential step in estimating environmental exposure [2,3]. Investigators in this area, particularly when involving several countries in multi-center studies, are faced with a high diversity of diets, depending on geographical area, cultural habits, education, socioeconomic components, and migration of minorities into native populations.

Variations in dietary branched-chained amino acids (BCAA), which consist of leucine, isoleucine and valine, were shown to partially mediate some of the benefits on decrease in Body Mass Index (BMI) or fat mass in both rodents and humans [4,5,6,7,8,9,10,11]. Dietary intake of BCAA might contribute to their plasma levels. Rietman et al. suggested that most dietary BCAA (80%) enters the bloodstream although other studies indicated that this percentage was <40% and presented weak correlations between BCAA intake and plasma levels [12,13,14,15,16]. Since the studies of Felig et al. [17] the relationship between plasma levels of BCAA and obesity gained renewed interest because of the metabolomics studies by Newgard et al. that demonstrated BCAA’s role in insulin resistance [18]. Thus, plasma BCAA levels are now considered as metabolic signatures of “diabesity” or MetS and are used as potential biomarkers for the development of diabetes [16,19,20,21,22,23]. In humans, high BCAA intake has been associated with an increased risk of T2D and insulin resistance incidences [15,24]. However, both long- and short-term interventional studies involving lean or obese populations showed rather contradictory results [25]. In all cases, accurate estimation of BCAA dietary intake is an absolute requirement.

BCAA intake was also included as part of the working hypothesis in the European MEDIGENE (standing for Mediterranean Genes) program (FP7-279171) that focused on MetS and long-term complications in Mediterranean countries (https://cordis.europa.eu/project/rcn/101810_en.html). MEDIGENE investigators were also faced with a wide variety of food items and recipes in addition to language barriers [26,27]. In dietary investigations, both the Food Frequency Questionnaire (FFQ) and 24-hour dietary recall (24HDR) recently benefitted from innovations in computer science, including food pictures, camera and tape recordings, or food bar code readers [28,29,30]. Several computer programs have emerged as both interactive computer- and web-based technologies [31,32]. The best known are US Department of Agriculture (USDA) automated multiple pass method, the University of Minnesota’s nutrition data system for research, computer-assisted 24HDR method (EPIC-soft) developed in the frame of the European Prospective Investigation into Cancer and Nutrition study and the Oxford WebQ [33,34,35,36,37]. In France, the NutriNet initiative was applied for wide ranges of human diseases, including MetS [38].

To solve problems raised by the variability of more than 11 countries in the MEDIGENE program and to efficiently estimate BCAA contents in food items, the objective of this study was to develop a new BCAA database embedded in a software system named MEDIPAD (standing for Mediterranean iPAD tablet computer) that can manage clinical, nutritional, and genetic anthropological data (https://cordis.europa.eu/result/rcn/195753_fr.html). The program was written in partnership with Intactile Design SA (France) and replaced the former Système Informatique Diététique Intégrée (SIDI) software developed by P Than Chi and L Monnier (Lapeyronie Hospital, CHU, Montpellier, France) [39]. Since MEDIPAD offers new facilities for food administration, in this paper we briefly present the new program and how we developed the BCAA database together with the evaluation of applicability of this program on 4559 24HDR from Saint Pierre Institute (SPI) in Palavas (France), and 119 available surveys from MEDIGENE in Mediterranean countries.

## 2. Materials and Methods

### 2.1. Computer Program

**Figure 1:**
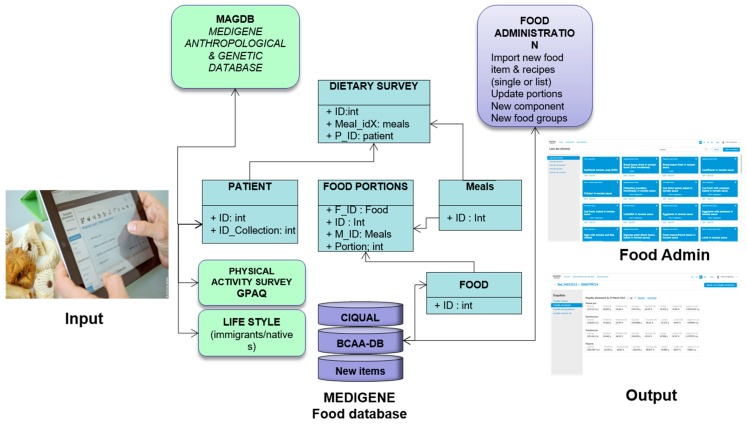
General architecture of MEDIPAD software.

For 24HDR collection, the new software package MEDIPAD was written using PHP (http://www.php.net) and MySQL (http://dev.mysql.com) for the database. It is composed of several functional interacting blocks as shown in Figure 1. The dietary record block has been linked with the database with clinical, biological, and genetic data and has been designated MEDIGENE Anthropologic and Genetic Database (MAGDB). The practitioner can access the program from an iPAD or laptop computer via MEDIGENE portal using username and password and is thus able to securely introduce new patient data from dietary surveys into the database. 

To introduce new food items, the following information is required by the administrator: (1) name of the food/recipe in French, English, Spanish or Romanian; (2) description of the item and comments if necessary; (3) food group according to MEDIPAD or other groups created by the administrator; and (4) food portion found in the database or created by the administrator.

The nutritional composition of each item is expressed in 100 g of edible portion. During the nutritional assessment, the dietitian can introduce a new food item or recipe that is not present in MEDIPAD by depositing a provisional written comment and/or a full description of ingredients and preparation procedures. The administrator can then filter and check added items, validate, and complete registration with the nutritional composition and related information. For mixed-dish items, the nutritional composition was calculated with the former SIDI software (this action is not allowed in MEDIPAD). In contrast, The MEDIPAD program permits creation of new food groups. For this aim, the administrator should provide the name of subproject in the consortium and food groups. In a second step, the administrator should assign each food item in the database to a food group. This last function could also be used, for instance, for the estimation of BCAA intake in food groups. The administrator can also add new users, validate patients or dietary surveys introduced by users, import new series of patients (as a table), or export nutritional analysis as .csv files. For analysis, MEDIPAD can calculate 61 components in foods as output, but there is no statistical treatment available in the actual version. The output parameters include macro-elements (such as sodium, magnesium), trace elements (such as zinc, selenium), amino acids, fatty acids, fat and water soluble vitamins, organic acids, sterols, total protein (g/100 g), carbohydrate (g/100 g), sugars (g/100 g), starch (g/100 g), polyols (g/100 g), fibers (g/100 g), water (g/100 g), total fats (g/100 g), including saturated and unsaturated fats, and alcohol consumption (g/100 g). With the compilation of BCAA in foods, the program can calculate isoleucine, leucine, valine, and total BCAA (defined as the sum of individual amino acid and expressed as g/100 g food portion).

### 2.2. BCAA (Branched-Chain Amino Acids) Database

The database was developed based on the French (CIQUAL) dietary tables (https://pro.anses.fr/TableCIQUAL/index.ht). Because this database does not contain amino acids in food composition and for MEDIGENE goals, BCAA content in foods was compiled for 1331 food items from the 2012 CIQUAL version. The strategy of the compiler was similar to that proposed by Suga et al. [40] based on similarities in food items by name with the description and nutrient composition and, in particular, taking the protein content into account. Nutrient tolerances per 100 g of food were used to estimate how close is a food item to the reference. For protein, nutrient tolerances were considered ±5 g [41]. The nitrogen value was estimated by dividing protein content by the nitrogen-to-protein conversion factor. For new items, we used the crude protein (N × 6.25) from CIQUAL while for the reference items, protein content with the corresponding nitrogen-to-protein conversion factor was used. The strategy of compiling consisted of several methods:

**Method 1.** Similarities were searched in other food table compositions, in particular from USDA food composition tables (https://ndb.nal.usda.gov), Danish Food Composition Databank (http://www.foodcomp.dk) and Canadian Nutrient File (https://food-nutrition.canada.ca). Amino acid values were adjusted considering differences in the nitrogen (N) of each food item using the following equation [40]:

*BCAA value of food in question* = *BCAA value of reference food* × *N of food in question*/*N of reference food*

**Method 2.** When data were not found in international food composition tables, we considered published data in which food items have been analyzed by high-performance liquid chromatography (HPLC) or gas chromatography (GC).

**Method 3.** When data were not available from the above described methods, we used the similarity of foods procedure. When the amino acid value was available for the raw food item, we estimated the amino acid value of cooked food. For nutrient loss or gain during cooking, we considered that the amino acid ratio was not modified but adjusted values for cooked food according to differences in nitrogen values using the following equation [40]:

*BCAA value of food in question (cooked)* = *BCAA value of reference food (raw)* × *N of food in question (cooked)*/*N of reference food (raw)*

Another way of estimating amino acid content by similarity was to use values from another food item of the same family. In this case, the amino acid content was adjusted and differences in N value were corrected using the same equation as described above.

**Method 4.** We also calculated the BCAA content in mixed dishes. Descriptions of the food items and ingredient lists were not available in all cases; in these cases, we have searched for similar recipes and determined the proportion of ingredients in 100 g food. We took cooking-related losses into account and substituted the corresponding quantities of raw ingredients with cooked ones. At the end, BCAA content in the mixed dish was obtained by summing the content of ingredients.

**Method 5.** Zero content for each isoleucine, leucine and valine was assumed in food items that do not contain proteins.

Unless indicated, items were assigned to ten food groups: (1) Dairy products and cheese; (2) Vegetables, fruits; (3) Cereals and pasta, (4) Meat, poultry, and fish; (5) Sugars and confectioneries; (6) Fats and oils; (7) Beverages; (8) Sauces and condiments; (9) Mixed dishes and soups; (10) Items for particular nutritional uses. For a better analysis of BCAA content we broke down these food groups into 18 groups as follows: 1—Offal; 2—Red meat and poultry; 3—Luncheon meats; 4—Fish and seafood; 5—Milk and dairy products; 6—Cheese; 7—Eggs and related products; 8—Bread, Pasta, cereals; 9—Pastries and brioches; 10—Cakes; 11—Fruits and vegetables; 12—Legumes; 13—Nuts and Seeds; 14—Mixed Dishes; 15—Fats and oil; 16—Sugar and confectionery; 17—Drinks; 18—Herbs, spices and condiments.

### 2.3. Populations and Ethical Statement

Applicability of MEDIPAD program with the BCAA database was evaluated on dietary surveys from 119 subjects in the MEDIGENE program and 2957 subjects from archives of SPI. The study design was cross-sectional and for this study we stratified patients by function of dietary intake of BCAA (quartiles) and by function of BMI (normal weight and obese). In the SPI survey, patients were divided into three groups based on growth charts (0–18 years old) from the Programme National Nutrition Santé (PNNS) (http://inpes.santepubliquefrance.fr/CFESBases/catalogue/pdf/IMC/courbes_enfants.pdf): (1) normal body weight; (2) overweight; and (3) obese and in some cases regrouped in two categories: Normal weight and obese. In MEDIGENE adult populations, the BMI cut-off value for obesity was 30 kg/m^2^. 

National Ethical Committees approved the study protocols, and informed consent was obtained from each patient in accordance with the Helsinki Declaration [42]. All collections were reported to the Ministère de l’Enseignement Supérieur de la Recherche et de l’Innovation (MESRI) in France under CODECOH # DC-2014-2226.

Populations recruited during MEDIGENE program (2012–2016) was multiethnic and included patients from France, Greece, Turkey, and Romania for which we had available 24HDR (*n* = 128) registered in MEDIPAD and performed by a professional dietitian via the MEDIGENE portal. As standard, we registered one week day, one weekend day and the day before the consultation. Patients were recruited for MetS studies, and diagnoses were based on the National Cholesterol Education Program (NCEP) and Adult Treatment Panel-III (ATP-III) criteria [43] as the presence of three or more of the following criteria: waist circumference, triglyceride and High-density lipoprotein (HDL) levels, the presence of blood pressure, hyperglycemia or medication for the last four criteria.

Populations for the achieved dietary records (*n* = 4559) of SPI (Palavas, France) concerned children and young adults of 3 to 24 years (*n* = 2957) investigated for obesity since this institute is specializes in obesity care in young adults and children. The dietary investigation was obtained by face-to-face interviews with a specialized dietician. As standard, all records concerned one day at the time of hospitalization. For many records we also used an additional week day and another weekend using the SIDI program (P. Garandeau and P. Than Chi) between 2007 and 2011 [39]. From the entire collection, for this study the type 1 diabetic patients were excluded, and for the remaining patients, we used available data on age, sex, and BMI values (kg/m^2^), but no data were available on insulin resistance or MetS. No follow-up studies were performed in the investigation. 

### 2.4. Dietary and Physical Activity Assessment

The MEDIPAD nutritional software can collect 24HDR face-to-face interview data by trained personal (dietician/physician) for one to three days via a web connection with the MEDIGENE server. During the interview, a patient is questioned about the quantity of food items with reference to the food portions during each meal: (1) breakfast; (2) lunch; (3) dinner and (4) snacks (screenshots are illustrated in Figures S1 and S2). The administrator has access to the clinical database and can anonymously collect clinical and dietary data in addition to any clinician’s comments. The general architecture of the nutritional block is depicted in Figure 1.

To assess physical activity levels, the global physical activity questionnaire (GPAQ) was introduced in French, English, and Spanish [44]. The GPAQ is composed of questions concerning: activity at work, travel to and from places, leisure activities and sedentary behaviors (total 16 questions). Questionnaire answers and results such as the total physical activity level, Metabolic Equivalent of Task (METs) in min and h per week can be exported in .csv file for additional statistics. The analysis was based on World Health Organization (WHO) guidelines [45]. The interviewer can then introduce physical activity levels (low, moderate, intense) and clinical data.

An additional feature of MEDIPAD is the delivery of a nutritional assessment report at the end of an interview. Each patient receives a summary of his/her nutritional status, actual caloric intake, and caloric needs. The estimated daily caloric needs can be calculated according to physical activity level and age. Macronutrient intake, vitamins, and minerals are also provided with recommended daily intake using ANSES (French Agency for Food, Environmental and Occupational Health & Safety) as the reference [46]. A summary questionnaire concerning socioeconomic and demographic data, dietary habits, lifestyle factors, and changes in dietary habits during immigration were also included.

Data derived from SPI were introduced in MEDIPAD using MySQL and Python scripts. Under- and over-reporters of energy intake were excluded based on physical activity levels assessed by the GPAQ also included in MEDIPAD [46]. For missing data on physical activity, outliers of energy intake were considered >4000 Kcal and <1000 Kcal. Basal metabolic rate (BMR) was calculated using the Harris & Benedict formula [47].

### 2.5. Data Management and Statistical Methods

Nutritional analysis was processed through MEDIPAD as 24HDR and exported in .csv file for further statistical analyses. For each food item in dietary records, we considered whether the matched food item presented in both MEDIPAD and CIQUAL. The statistical significance level was considered at *p* < 0.05. Numerical values were expressed as mean ± SEM and when indicated for nonparametric analysis we used Mann Whitney or Kruskal-Wallis test. For analysis of BCAA values we used analysis of variance (ANOVA) and logistic regression comparing groups of patients composed by quartiles (Q1, Q2, Q3, Q4) of total BCAA (Branched-Chain Amino Acids) intake and using several models (adjustment for age and energy intake). Statistical significance for logistic regression was presented as *p*-value with odds ratio and 95% CI (Confidence Interval). For ANOVA we used fixed the 2α level at 5% as previously described [48]. All statistics were performed using Statview 5.0 (SAS, Abacus Concepts, Berkeley, CA, USA) and JMP 13 (SAS Institute Inc., Cary, NC, USA, 1989–2007). 

## 3. Results

### 3.1. Description of BCAA Database

For the BCAA database, the chief section in the MEDIPAD program is the section for food administration (Food Admin). Through this interface, the administrator is able to obtain the list of food items, groups, and food composition and can manage the following additional functions: (1) manage the list of portions; (2) check the list of nutritional components; (3) create new food groups for research purposes; (4) manually add new food items or recipes; and (5) import food items as bulk in .csv file (screenshots are illustrated in Figure 1).

The French CIQUAL database 2012 containing 1331 food items was updated with up to 2137 food items by adding several parameters:

1. Food items (*n* = 430) were completed from previous observations in SIDI program between 1992 and 2012 as compiled by Dr. P. Than Chi (Lapeyronie Hospital, Montpellier, France).
2. For the BCAA database, the chief section in the MEDIPAD program is the section for food administration (Food Admin). Through this interface, the administrator is able to obtain the list of food items, groups, and food composition and can manage the following additional functions: (1) manage the list of portions; (2) check the list of nutritional components; (3) create new food groups for research purposes; (4) manually add new food items or recipes; and (5) import food items as bulk in .csv file (screenshots are illustrated in Figure 1). Greek food items and traditional dishes (*n* = 141) were added from the composition table of foods and Greek dishes of the Hellenic Health Foundation (http://www.hhf-greece.gr/tables/home.aspx?l=en). The source was “Composition tables of foods and Greek dishes”, 3rd edition [49].
3. Turkish food items and dishes (*n* = 72) were included from Turkish Food Composition Database (TürKomp) (http://turkomp.gov.tr/database?type=foods), and recipes calculated from Turkish cuisine had been developed by the Ministry of Health (General Directorate of Primary Health Care & Primary Healthcare Department of Nutrition and Physical Activity) [50].
4. Romanian (*n* = 22) and Moroccan (*n* = 32) dishes (mixed-dishes) were also added and calculated in SIDI program using the National Composition Table of foods available for Romanian foods (http://hunkbody.ro/tabel-cu-continutul-nutritiv-al-alimentelor/).

Repartition of food items such as the method’s function used for BCAA content in CIQUAL along with the number of items in each group are indicated in Table 1. Several the 1331 items were compiled and the majority (75%) came from other food composition tables. Thus, a total of 993 items were compiled using method 1, seven items using published analytic measurements, six items from method 3, and 174 items were from calculated recipes. Of note, 151 food items were assumed to have zero content, and 109 items were not compiled. The mean BCAA content (g/100 g) classified by food groups is indicated in Table S1. The meat/poultry/fish and dairy products/cheese groups had the highest content of BCAA (3.62 ± 0.06 g/100 g and 2.37 ± 0.07 g/100 g, respectively). For different countries, we included only food items that were added in MEDIGENE surveys. For Turkish food items, BCAA content was extracted from TürKomp or from recipes. For Greek and Romanian dishes, BCAA content was calculated exclusively from recipes (Moroccan surveys were not finalized).

### 3.2. Food Items as Function of BCAA Content

To validate the soundness of the database’s compiled food items, we used a repertoire of 4559 surveys from SPI and compared those surveys to the entire CIQUAL database. Food items were stratified as functions of BCAA content percentiles (g/100 g) as indicated in Table 2 and Figure S3. Six categories were generated: (1) A: zero content; (2) B: 0–0.232; (3) C: 0.233–1.083; (4) D: 1.084–2.658; (5) E: 2.659–4.168; and (6) F: >4.168 g/100 g. Percentiles of CIQUAL food items were in accordance with those used in the large SPI repertoire. Thus, in the first category (A), we mainly find the food groups without proteins such as fats and oils, sugar or confectioneries, and drinks. In the last category (F), we found items with the highest BCAA content consisting of animal products with the several food subgroups: (1) cheese (30.41%); (2) red meat and poultry (29.73%); (3) fish and seafood (17.57%); (4) lunch meats (7.43); and (5) organ meats (5.41%).

### 3.3. Major Contributors to BCAA Intake in SPI Surveys

In the next step and to further validate our new database using population data, we focused on SPI surveys considering age categories from 9 to 13 and from 14 to 18 years, stratified by BCAA quartiles. After exclusion of over- and under-reporters along with aberrant values or missing data, we had 24HDR from 2957 subjects. Two-thousand, one-hundred and seventy-five (1161 females/1014 males) and 782 (501 females/281 males) subjects were included in the age groups of 9 to 13 and 14 to 18 years, respectively. Their clinical profiles are indicated in Table S2. When the intake of total BCAA and isolated amino acids were studied by logistic regression using the stratification of values by quartiles, subjects at the highest quartiles of BCAA intake had proportional in the OR of association with obesity (Table 3). However, the statistical significance was obtained only for isoleucine and total BCAA in age group 9–13 years. 

By using the method of Block et al., we next estimated the contribution of BCAA intake to 18 food groups [51]. The major contributors to total BCAA or independent amino acids (isoleucine, leucine, and valine) are listed in decreasing order: (1) red meat and poultry; (2) bread; (3) pasta and cereals; (4) milk and dairy products; (5) cheese; (6) legumes; and (7) lunch meats (Table 4). The relative contribution applied to dietary surveys in SPI in subjects in both age groups and stratified by gender and BMI are indicated in Tables S3 and S4. 

### 3.4. Evaluation of BCAA Intake in MEDIGENE Compared to Other Studies

A second application of the database was performed to compare data from MEDIGENE to other studies in the literature that used a similar methodology. Clinical profiles of these patients with a diagnosis of MetS are indicated in Table S5. In brief, there were no statistical differences between females and males for BMI, insulin resistance and other components of MetS. The energy intake was higher in men than in women (*p* < 0.006, Mann Whitney). The mean BCAA intake in MEDIGENE collection was compared between normal weight and obese subjects. In females, obese subjects had a higher intake of isoleucine, leucine, and valine compared to normal weight subjects (*p* < 0.0328, <0.0071, and <0.052, respectively). After adjusting for energy intake, significance was lower for leucine (*p* < 0.0961) and lost for isoleucine (*p* < 0.2327). In men, no differences were found between normal weight and obese subjects’ intake (Table S6).

## 4. Discussion

In this paper, we described a new database with BCAA content in food items. This database may be used for dietary surveys using 24HDR as part of the novel computer software in the MEDIGENE European program for the study of insulin resistance in Mediterranean countries. The MEDIPAD program is advantageous as it helps clinicians or researchers enter clinical, biological, and 24HDR for Mediterranean countries. The project was initiated with the goal of studying Gene × Diet interactions in the pathogenesis of insulin resistance. So far, the major advantage of the program was the new section of food administration that can manage data for mixed-dish recipes, modifying items as a function of country and language, and providing a contact with investigators in various countries. The Mediterranean area displays diverse dietary habits and data collection based on many different food databases more or less specific to countries is a difficult task.

The necessity of MEDIPAD program emerged from the multitude of countries and clinical hospitals that were involved in this study but also from new aspects of immigration of ethnic groups into another county (such as Albanians into Greece) and areas in which dietary surveys should be performed in native and immigrant populations. The European MEDIGENE project involved Mediterranean countries (Spain, France, Italy, Greece, Croatia, Albania, Romania, Turkey, Russia, Algeria, Morocco, and Tunisia) and was intended to study genetic and environmental risk factors involved in the pathogenesis of insulin resistance syndrome. Therefore, the best way to harmonize the dietary data collections was to create a program common to all countries with a unique database, including a database for food items. To help the interviewer, we conceived a web-based design that can introduce data from countries using a laptop computer or iPAD on a unique server implemented in France. The MEDIPAD requires a secure web (https) connection that allows entry of nominal data. At present, the program benefitted from a specialized interviewer, a dietician, or a nutritionist in addition to the contribution of a food administrator for completion of the database, manipulation, and standardization. MEDIPAD was written in French, English, Spanish, and Romanian (Arabic is missing in the actual version). Informatics administration is under the responsibility the Département de Systèmes d’Information of Montpellier University (France) at which a dedicated IT staff assured conditions for archiving and data backup.

In this project, we focused on 24HDR since multiple recordings on several days are frequently used as reference nutritional instruments for accurate dietary assessments in connection with metabolic disorders [33,52]. The 24HDR was also the method of choice because it does not require computer literacy, is cheap, and the interview is time short (<20 min). However, the procedure is not free from errors and biases. As for other procedures, errors may come from food reporting, food identification, and food quantitation [52].

In many aspects, the program is similar to several other computer programs described in the literature using a 24HDR [33,35,36,53]. Very close to our software are, for instance, Assisted Personal Interview System (CAPIS) in Korea, NINA-DISH, EPIC-SOFT, DietDay [35,54,55,56,57]. Design and validation of web- and computer-based 24HDR were recently review in Ref. [33]. Thus, the program uses a friendly graphical interface (icons for aliment groups) and pull-down menus to help with the input of diet data. There are 61 parameters calculated by the program, including protein, lipid, and sugar consumption. We have adhered to the CIQUAL database in France as the baseline to which we added 697 food items from the former program SIDI or other databases. Currently, the database is improving and includes the 2016 new CIQUAL version. The food administrator can match a new item from CIQUAL with MEDIPAD database (containing the ORIGFDCD attribute, which represents the food codes in CIQUAL) and add the new item manually or via the .csv files. Although not used in this study, another advantage of the MEDIPAD is the ability to connect patient data for dietary records with the MEDIGENE Anthropological and Genetic DataBase (MAGDB) containing genetic, anthropological, and biological information. In contrast, the program is unable to perform statistical calculations; therefore, external programs should analyze exported data. For this aim, MEDIPAD contains facilities for exporting data as .csv files, which can be easily introduced in current statistical programs. MEDIPAD does not contain graphical displays for sizes and portions of dishes although that issue is important. Therefore, a specialized dietician or nutritionist should introduce the data. For mixed dishes, we used the former SIDI program, but this function will be soon available in accordance to algorithms that will complete the program in nutritional counselling.

As shown in this study, the major advantage of MEDIPAD is the possibility to add additional nutrients by compilation of BCAA content of food items in a database, and thus allowing to investigate efficiently the role of BCAA or to test hypothesis of pathogenesis of insulin resistance. Recent studies have indicated that not only the evaluation of total protein or total BCAA in the diet is crucial in understanding gene diet interaction, but precise composition of diet for each BCAA is an important determinant of metabolic status [10]. Along this line, particular “patterns of food” items appeared to be related with MetS [58] and in particular case of BCAA with addition long-term use [14]. In another study, in France, it was also indicated that for adequate protein and amino acids intake, animal/plant protein ratio in the diet requires changes in the pattern of plant protein intake [59]. We considered that flexibility in creating and changing database for food items would be a crucial advantage of a new computer program for nutritional analysis. Therefore, we designed a specific function of food administration. Using the same function, one can introduce other components such as polyphenol content and glycemic and insulinogenic indices of foods. Along this line, another advantage of MEDIPAD is the possibility to modify or create new food groups as a function of various scientific hypotheses in the literature and to export nutritional analysis as function of food groups.

For the compilation of BCAA, most food items were obtained from other international food composition tables, except for 109 items that were not compiled. By using five methods, we were able to compile 1331 items, among which 75% was obtained from other alimentary tables. Before publication of this database following the FAO/INFOOODS criteria, we showed that application of the new BCAA database for available records yielded comparable results [60]. Thus, the repertoire of 4559 24HDRs was similar to that of the entire CIQUAL database, including the food items with the highest BCAA content. Similarly, BCAA intake estimations in normal weight and obese individuals or separated by gender were comparable to the results from other studies in Japan (Table S7). To compare to other studies ranges of age were fixed between 27 and 65. After excluding subjects >65 years, we obtained up to 119 available surveys. BCAA mean intake was higher in men than in women (13.84 versus 12.91 g/day) representing a 7% increase. Ishikawa-Takata and Takimoto [61] also reported higher BCAA intake in the same age category (30–64 years) with a mean of 17.20 and 14.70 g/day in men and women, respectively, representing a 15% increase. Similar higher intake in males (16.10 and 16.59 g/day) was reported by Ishihara et al. [62] in another two cohorts. In men, the mean intake was close to the MEDIGENE data and that of Suga et al. [40,63] using a similar BCAA compiled database (Table S7). No major differences were found among ethnic groups in MEDIGENE surveys. Our data were also comparable to another recent BCAA study concerning the contribution of food groups to the content of each amino acid [59]. Our study was also comparable with that of adolescent residents in Brazil from the Health Survey of São Paulo concerning unprocessed red meat, poultry, savory baked, bread and toast, and beans and rice as contributors to BCAA intake [64]. In the US population, the major contributors were shown to be meat, milk, and fish [15]. However, in our population, in which the age range was different from the US study, fish and seafood had a lower contribution of 2% similar to the São Paulo study [64]. Also, we should mention differences in dietary habits in each country. For instance, in Japan, cereals/potatoes and starches are the main contributors to BCAA intake followed by fish and meat [65], and in China, the major contributors are rice, wheat foods, livestock and its products, milk, and eggs [66].

Most food items were compiled from other food composition tables. The limitation of this methodology is obvious for mixed dishes in which we had to use similar recipes to calculate the BCAA content, except for Greek dishes in which the recipes were described. For other specific country dishes (such as Turkish, Moroccan, Romanian) BCAA was calculated solely from the recipes. One of the other study limitations was the BCAA content in cheese. CIQUAL contains a large variety of cheeses not found in other tables. In these cases, a similar type of cheese was substituted. The contribution of food groups to BCAA intake was calculated for the SPI collection to have representation in the French collection, and indeed our results were similar to others reported in the literature.

At this stage of the development, this study has several limitations, particularly related to the validation of the BCAA database and potential correlation between the dietary intake and plasma levels of amino acids. Indeed, the database should be validated based on guidelines of FAO/INFOODS [60] including tagnames for food items, criteria for establishing recipes and mixed dishes. In next steps, several tools might be used such as compilation tool FAO/INFOODS 1.2.1. Since most food items are coming from international databases and publications data at this stage there are no reasons to doubt about the accuracy of data. However, a special action of checking will be performed in the future. Another important issue is that the coefficient of variation for amino acids in food groups are approximately homogenous, which ideally should be kept between 10 and 20%. Along this line, database quality will be assessed in the future using several progressive grades and scoring. Another aspect that should be considered in perspective is the potential correlation with plasma levels of BCAA at various intake levels. Finally, there are several future improvements expected for the program itself. One major feature is obviously the advice component, which depends on the algorithm to be used. In collaboration with the Bioinformatics Department of the University of Montpellier, preliminary work was initiated for constraint programming. Such an algorithm will not solve the difficult problem of menu planning but can be useful in determine BCAA content in food repertoires in rare cases of inborn errors of metabolism due to genetic defects in BCAA catabolism specific for each of the three BCAA [24]. Other features to be considered in the near future include the possibility of the administrator calculating recipes similar to the former SIDI program, use of more languages such as Arabic, which would be useful in North Africa, and adaptation of the program for smartphones.

## 5. Conclusions

In conclusion, MEDIPAD and its associated BCAA database provide a useful integrated software system that help to handle acquisition, evaluation, and compilation of food composition data and is applicable in the Mediterranean area. Besides BCAA plasma measurements in research studies, the program is an obligatory step for the accurate estimation of BCAA intake for further nutrigenomic investigations. Accessible from iPAD or laptop computer and already written in four languages, the program uses a friendly interface, which is useful for practitioners since is web-linked and secure, thus facilitating storage of data in France on a server with secure archiving (everyday) system and backup (each week) at the university site. MEDIPAD offers numerous possibilities for data extraction for statistical analysis and may be completed with new databases for food items. In conjunction with other European software in the same field, MEDIPAD represents valuable output from the MEDIGENE European program with applicability in Southern countries. In perspective, after the validation of the database complying with FAO/INFOOD criteria [60], we intend to develop the program for the application in nutrigenomics studies taking into consideration both nutritional intake and genetic susceptibility. 

## Figures and Tables

**Table 1:** Number of food items compiled for BCAA database as function of methods used and food groups. Food items were assigned to different food groups and BCAA content was compiled using five methods (Non-compiled food items are also indicated).

Food Groups	Method 1	Method 2	Method 3	Method 4	Method 5	Total Compiled	Not Compiled
Dairy products and cheese	190	0	0	6	0	196	0
Vegetables, fruits	177	3	2	5	0	187	22
Cereals and Pasta	178	0	1	14	0	193	11
Meat, poultry and fish	248	4	3	13	0	268	5
Sugars and confectionery	28	0	0	4	2	34	4
Fats and oils	16	0	0	0	25	41	3
Beverages	49	0	0	8	117	174	33
Sauces and condiments	38	0	0	13	7	58	7
Mixed dishes and Soups	67	0	0	111	0	178	16
Items for particular nutritional uses	2	0	0	0	0	2	8
Total	993	7	6	174	151	1331	109

Method 1: Other food table composition; Method 2: Literature (analytic measurements); Method 3: Similar food items; Method 4: Calculated recipes; Method 5: Assumed zero content.

**Table 2:** Food groups distribution per centiles of total BCAA content.

Food Groups (%)	A	B	C	D	E	F
Offal	0.00	0.00	0.00	2.42	4.95	5.41
Red meat and poultry	0.00	0.00	0.00	1.34	25.68	29.73
Luncheon meats	0.00	0.00	0.00	7.80	13.51	7.43
Fish and seafood	0.00	0.00	0.54	5.38	30.18	17.57
Milk and dairy products	0.00	0.99	24.12	5.65	3.60	5.41
Cheese	0.00	0.00	1.08	6.45	7.21	30.41
Eggs and related products	0.00	0.00	0.00	1.61	4.05	0.00
Bread, Pasta, cereals	0.00	0.49	14.36	24.46	0.45	0.68
Pastries and brioches	0.00	0.00	1.63	3.49	0.00	0.00
Cakes	0.00	0.49	13.82	3.76	0.00	0.00
Fruits and vegetables	1.78	50.25	16.80	0.54	0.00	0.00
Legumes	0.00	0.00	1.63	2.42	0.90	0.00
Nuts and seeds	0.00	0.49	1.90	2.96	3.60	0.68
Mixed dishes	0.59	6.90	10.03	26.34	4.50	1.35
Fats and oil	15.98	5.91	1.36	0.00	0.00	0.00
Sugar and confectionery	3.55	5.42	2.44	2.96	0.00	0.00
Drinks	73.96	20.69	3.52	0.27	0.90	0.00
Herbs, spices and condiments	4.14	8.37	6.78	2.15	0.45	1.35

Centiles are represented as content of BCAA in g per 100 g of food portion; A, zero content; B, 0–0.232; C, 0.233–1.083; D, 1.084–2.658; E, 2.659–4.168; F, >4.168.

**Table 3:** Relationship between quartiles of BCAA intake and obesity in SPI collection stratified by age category.

Variable	Q1	Q2	Q3	Q4	*p*
**Isoleucine**					
Median	3.67	4.46	5.18	6.17	
Obesity (9–13) ^a^	1.00	1.19 (0.91–1.55)	1.48 (1.11–1.96)	1.60 (1.14–2.24)	0.0198
Median	3.60	4.47	5.19	6.21	
Obesity (14–18) ^a^	1.00	0.63 (0.39–1.03)	0.96 (0.59–2.09)	0.92 (0.53–1.60)	0.1936
**Leucine**					
Median	5.97	7.27	8.48	10.14	
Obesity (9–13) ^a^	1.00	1.29 (1.00–1.68)	1.33 (1.00–1.75)	1.41 (1.01–1.97)	0.1330
Median	5.82	7.27	8.45	10.12	
Obesity (14–18) ^a^	1.00	0.76 (0.47–1.22)	0.86 (0.51–1.43)	0.90 (0.52–1.56)	0.7164
**Valine**					
Median	4.21	5.17	5.1	7.00	
Obesity (9–13) ^a^	1.00	1.21 (0.93–1.57)	1.29 (0.97–1.71)	1.57 (1.12–2.20)	0.0725
Median	4.14	5.12	5.96	7.04	
Obesity (14–18) ^a^	1.00	0.73 (0.45–1.17)	0.96 (0.57–1.60)	0.90 (0.51–1.57)	0.5141
**Total BCAA**					
Median	13.64	17.04	19.62	23.31	
Obesity (9–13) ^a^	1.00	1.30 (1.00–1.70)	1.33 (1.00–1.77)	1.61 (1.15–2.26)	0.0440
Median	13.64	17.04	19.62	23.31	
Obesity (14–18) ^a^	1.00	0.68 (0.43–1.10)	0.93 (0.56–1.54)	0.91 (0.52–1.58)	0.3883

^a^ Adjusted for age and energy intake. Quartiles for BCAA (g/100 g) were defined using the following cut-off values: 4.12, 4.8, 5.57 for isoleucine, 6.7, 7.89, 9.2 for leucine, 4.72, 5.5, 6.36 for valine and 15.6, 18.2, 21.1 for total BCAA for Q1, Q2, Q3 and Q4, respectively

**Table 4:** Contribution of food groups to total and individual BCAA intake in SPI collection.

Food Groups Contribution (%)	BCAA	Isoleucine	Leucine	Valine
Offal	0.02	0.02	0.02	0.01
Red meat and poultry	36.30	36.42	38.04	33.60
Luncheon meats	3.27	3.44	3.35	3.00
Fish and seafood	2.04	2.03	2.14	1.91
Milk and dairy products	16.72	15.97	16.69	17.52
Cheese	9.81	11.47	7.05	12.33
Eggs and related products	1.10	1.07	1.05	1.18
Bread, Pasta, cereals	17.30	16.67	17.86	17.03
Pastries and brioches	2.39	2.30	2.47	2.36
Cakes	0.64	0.64	0.63	0.64
Fruits and vegetables	1.72	1.84	1.75	1.56
Legumes	4.82	4.51	5.02	4.80
Nuts and seeds	0.05	0.05	0.05	0.05
Mixed dishes	1.76	1.69	1.83	1.73
Fats and oil	0.06	0.06	0.06	0.07
Sugar and confectionery	1.36	1.21	1.39	1.45
Drinks	0.53	0.51	0.48	0.64
Herbs, spices and condiments	0.11	0.11	0.10	0.10

## Supplementary Materials

The following are available online at http://www.mdpi.com/2072-6643/10/10/1392/s1, Figure S1: Screenshot of the MEDIPAD software illustrating the registration of 24HDR for the breakfast; Figure S2: Screenshot of the MEDIPAD software illustrating the registration of 24HDR for breakfast in the first day and possible corrections to assess quantity of foods; Figure S3: Distribution of BCAA content by centiles in CIQUAL French database and food items from SPI surveys; Table S1: Mean BCAA content (g/100 g) by food groups in 1331 compiled food items of CIQUAL; Table S2: Clinical features of patients from SPI collection stratified by BMI. Table S3: Contribution of food groups to total and individual BCAA intake in age category 9–13 years old in SPI surveys stratified by gender and nutritional status; Table S4: Contribution of food groups to total and individual BCAA intake in age category 14–18 years old in SPI surveys stratified by gender and nutritional status. Table S5: Clinical features of patients from MEDIGENE collection stratified by gender. Table S6: Mean intake of total and individual BCAA (g/day) stratified by BMI* in MEDIGENE surveys. Table S7: Mean intake of total and individual BCAA (g/day) stratified by gender in MEDIGENE surveys compared to other international studies.
