# Supplementary material for: Branched-Chain Amino Acid Database Integrated in MEDIPAD Software as a Tool for Nutritional Investigation of Mediterranean Populations
Source: Nutrients. 2018 Oct 1;10(10):1392. doi: 10.3390/nu10101392 (PMC6213539; doi:10.3390/nu10101392)
Supplement: Supplementary file 1 [file nutrients-10-01392-s001.pdf]

Supplementary material

Figure S1. Screenshot of the MEDIPAD software illustrating the registration of 24HDR for the breakfast

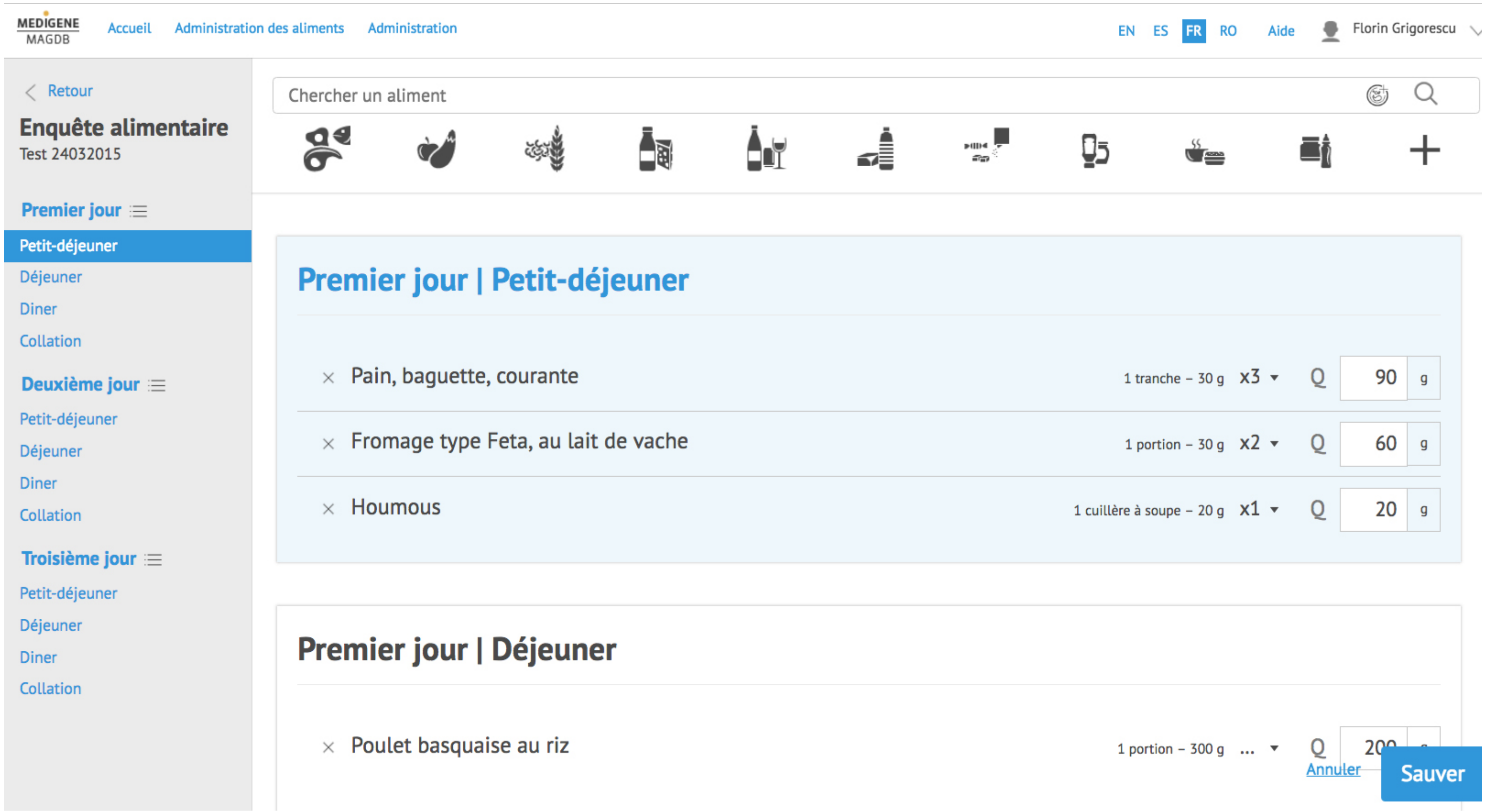

Figure S2. Screenshot of the MEDIPAD software illustrating the registration of 24HDR for breakfast in the first day and possible corrections to assess quantity of foods.

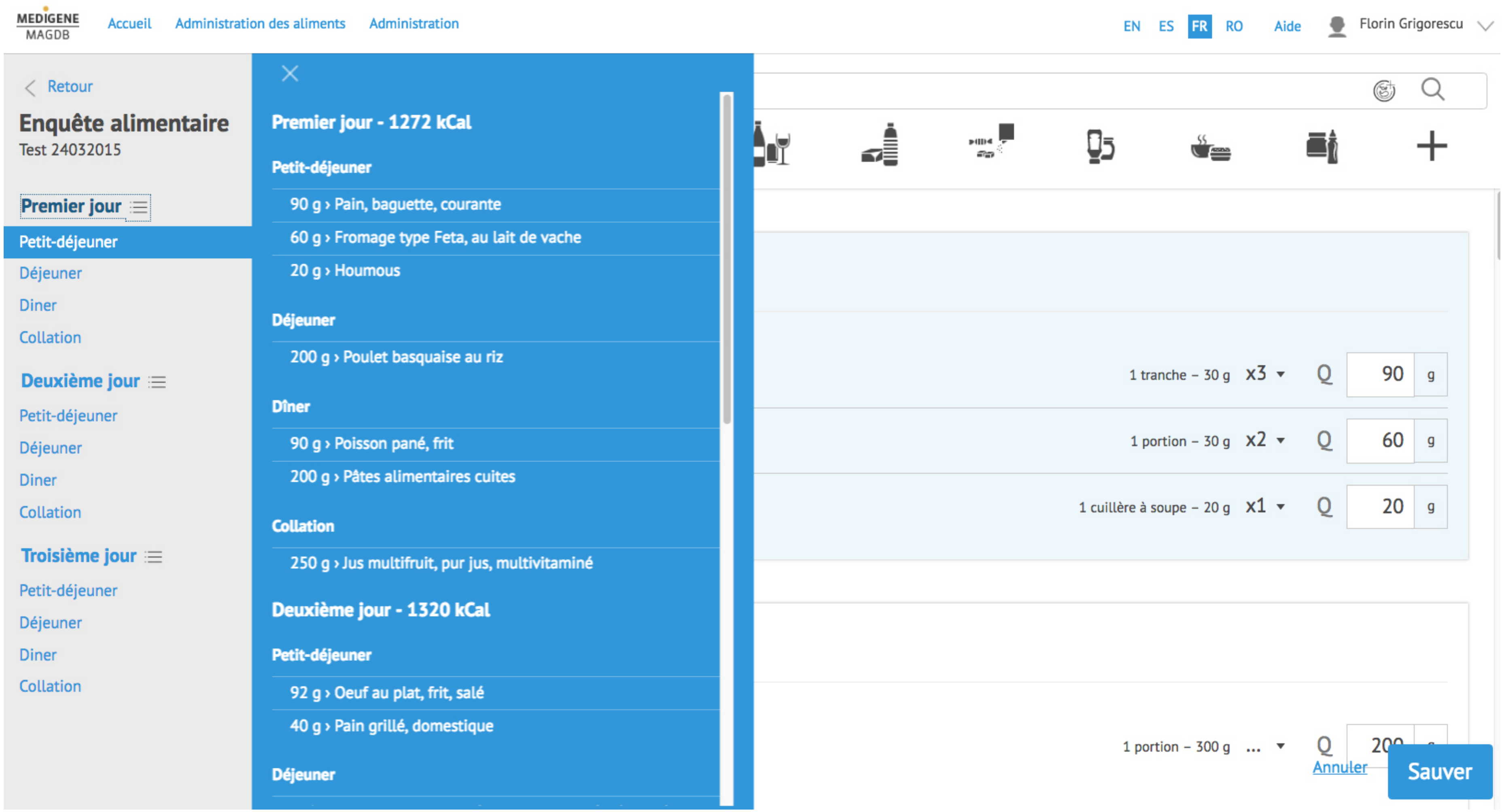

Figure S3. Distribution of BCAA content by centiles in CIQUAL French database and food items from SPI surveys

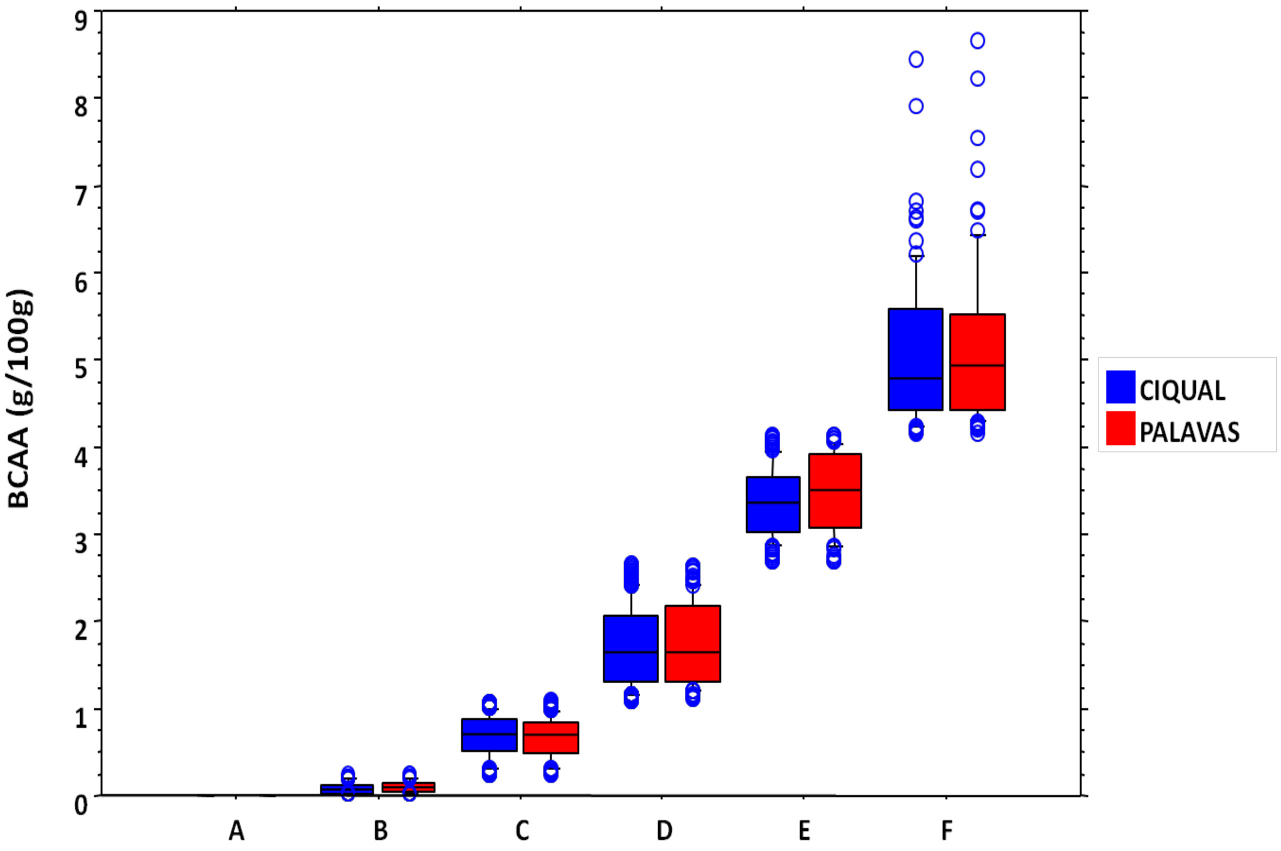

Table S1. Mean BCAA content (g/100 g) by food groups in 1331 compiled food items of CIQUAL

Food Groups	BCAA content
Dairy products & cheese	2.37 ± 0.07
Vegetables, fruits	0.50 ± 0.08
Cereals & Pasta	1.17 ± 0.07
Meat, poultry & fish	3.62 ± 0.06
Sugars & confectionery	0.61 ± 0.18
Fats & oils	0.08 ± 0.17
Beverages	0.06 ± 0.08
Sauces & condiments	0.68 ± 0.14
Mixed dishes & Soups	1.63 ± 0.08
Items for particular nutritional uses	0.54 ± 0.77

Values are expressed as Mean ± SE

Table S2. Clinical features of patients from the SPI collection stratified by BMI

	Normal weight	Obese	P-value (t-test)
n	1659	1298	NA
Age (years)	12.00 ± 0.05	12.16 ± 0.06	0.057
BMI (kg/m^2)	22.02 ± 0.09	30.55 ± 0.14	< 0.0001
Energy intake (Kcal/d)	2264.64 ± 12.14	2537.00 ± 15.33	< 0.0001
Protein intake (g/d)	102.95 ± 0.56	114.41 ± 0.68	< 0.0001
BCAA intake (g/d)	17.70 ± 0.10	19.56 ± 0.12	< 0.0001

Table S3. Contribution of food groups to total and individual BCAA intake in age category 9 – 13 years old in SPI survey stratified by gender and nutritional status.

Food Groups	Isoleucine						Leucine						Valine					
	Female NBW	Male NBW	Female OVW	Male OVW	Female OB	Male OB	Female NBW	Male NBW	Female OVW	Male OVW	Female OB	Male OB	Females NBW	Male NBW	Female OVW	Male OVW	Female OB	Male OB
Offals	0.00	0.00	0.05	0.00	0.04	0.04	0.00	0.00	0.05	0.00	0.05	0.05	0.00	0.00	0.05	0.00	0.04	0.00
Red meat & poultry	39.32	36.85	33.79	34.46	35.53	36.64	41.06	38.58	35.41	35.77	37.31	38.37	36.33	34.32	31.03	31.54	32.70	33.85
Luncheon meats	2.80	3.69	3.84	3.72	3.36	3.46	2.71	3.32	3.86	3.68	3.30	3.45	2.45	2.95	3.49	3.32	2.93	3.07
Fish & seafood	1.58	2.47	2.44	2.05	2.40	1.65	1.71	2.21	2.64	2.14	2.59	1.79	1.52	2.00	2.34	1.89	2.30	1.58
Milk & dairy products	15.49	16.30	16.63	17.17	15.47	16.23	16.16	17.09	17.40	17.88	16.29	16.94	17.19	18.11	18.19	18.71	16.92	17.70
Cheese	11.23	10.79	12.18	12.68	12.27	11.37	6.89	6.86	7.47	8.67	7.19	6.67	12.06	11.65	13.00	13.72	13.15	12.17
Eggs & related products	1.15	0.71	1.36	1.13	1.22	0.92	1.13	0.70	1.33	1.11	1.20	0.90	1.28	0.79	1.50	1.25	1.35	1.01
Bread. Pasta. cereals	16.86	18.79	15.63	16.11	16.35	16.70	17.97	20.11	16.75	17.22	17.68	17.93	17.24	19.30	15.87	16.39	16.71	17.06
Pastries & brioches	2.66	2.22	2.21	2.34	2.49	2.36	2.84	2.40	2.40	2.48	2.72	2.52	2.72	2.29	2.25	2.38	2.56	2.42
Cakes	0.70	0.61	0.76	0.56	0.60	0.48	0.69	0.62	0.76	0.56	0.61	0.48	0.70	0.62	0.77	0.56	0.61	0.48
Fruits & vegetables	1.94	1.87	2.00	1.70	1.79	1.54	1.99	1.81	1.91	1.62	1.72	1.45	1.66	1.62	1.69	1.44	1.52	1.30
Legumes	3.01	2.70	4.86	4.94	5.00	5.21	3.34	3.01	5.42	5.49	5.61	5.80	3.21	2.90	5.15	5.24	5.33	5.55
Nuts & Seeds	0.03	0.02	0.02	0.07	0.02	0.02	0.03	0.02	0.02	0.08	0.02	0.02	0.03	0.02	0.02	0.08	0.02	0.02
Mixed Dishes	1.54	1.22	2.17	1.04	1.39	1.57	1.69	1.30	2.36	1.10	1.49	1.68	1.62	1.27	2.23	1.05	1.42	1.59
Fats & oil	0.07	0.07	0.06	0.06	0.06	0.06	0.08	0.08	0.06	0.06	0.06	0.07	0.08	0.08	0.06	0.06	0.06	0.07
Sugar and confectionery	0.95	1.02	1.32	1.32	1.38	1.16	1.09	1.24	1.54	1.53	1.57	1.33	1.14	1.23	1.58	1.57	1.66	1.39
Drinks	0.64	0.60	0.56	0.57	0.51	0.47	0.58	0.57	0.51	0.54	0.47	0.44	0.74	0.77	0.69	0.72	0.63	0.60
Herbs, Spices & condiments	0.05	0.08	0.11	0.07	0.11	0.12	0.04	0.08	0.10	0.07	0.11	0.12	0.04	0.08	0.11	0.07	0.10	0.12

NBW, normal body weight; OVW, overweight; OB, obese

Table S4 Contribution of food groups to total and individual BCAA intake in age category 14 – 18 years old in SPI surveys stratified by gender and nutritional status

Food Groups	Isoleucine						Leucine						Valine					
	Female NBW	Male NBW	Female OVW	Male OVW	Female OB	Male OB	Female NBW	Male NBW	Female OVW	Male OVW	Female OB	Male OB	Female NBW	Male NBW	Female OVW	Male OVW	Femal e OB	Male OB
Offals	0.00	0.00	0.00	0.00	0.00	0.00	0.00	0.00	0.00	0.00	0.00	0.00	0.00	0.00	0.00	0.00	0.00	0.00
Red meat & poultry	37.59	40.54	37.04	39.33	38.04	38.82	38.99	41.77	38.59	41.59	39.57	40.42	34.74	37.66	34.28	36.56	35.20	35.95
Luncheon meats	2.98	3.31	3.88	3.58	2.91	3.12	2.49	2.87	3.50	3.42	2.94	3.20	2.18	2.58	3.08	3.09	2.67	2.87
Fish & seafood	2.79	0.37	2.45	0.52	1.99	1.38	2.97	0.39	2.66	0.56	2.14	1.45	2.70	0.36	2.35	0.50	1.92	1.34
Milk & dairy products	17.86	14.79	15.86	14.74	15.06	13.48	18.69	15.32	16.66	15.45	15.59	13.95	20.05	16.58	17.69	16.08	16.33	14.64
Cheese	9.33	9.03	10.57	11.31	11.25	10.03	5.82	5.91	6.59	6.23	7.17	5.68	10.00	9.86	11.54	12.07	12.25	10.74
Eggs and related products	1.53	1.25	0.73	1.01	0.63	1.00	1.49	1.21	0.72	0.99	0.62	0.97	1.70	1.39	0.82	1.11	0.70	1.11
Bread, Pasta, cereals	16.16	18.72	16.46	17.34	16.93	17.53	17.24	19.82	17.63	18.65	18.07	18.59	16.54	19.19	16.89	17.84	17.32	17.97
Pastries & brioches	1.69	1.88	2.16	1.60	2.37	2.41	1.80	2.04	2.30	1.71	2.53	2.55	1.74	1.90	2.24	1.65	2.44	2.48
cakes	0.72	0.74	0.47	0.68	0.70	0.91	0.72	0.73	0.47	0.68	0.69	0.89	0.72	0.76	0.47	0.68	0.71	0.92
Fruits & vegetables	2.31	1.73	2.40	1.50	2.13	1.54	2.18	1.62	2.24	1.42	1.97	1.44	1.96	1.45	2.02	1.30	1.81	1.34
Legumes	3.43	3.43	4.12	5.96	3.90	5.05	3.80	3.78	4.58	6.66	4.32	5.59	3.67	3.67	4.41	6.37	4.17	5.40
Nuts & Seeds	0.05	0.05	0.12	0.00	0.12	0.16	0.06	0.06	0.11	0.00	0.13	0.18	0.05	0.06	0.13	0.00	0.13	0.17
Mixed Dishes	1.94	2.63	1.78	0.89	2.17	2.64	2.08	2.89	1.92	0.98	2.32	2.93	1.99	2.72	1.84	0.92	2.20	2.73
Fats & oil	0.05	0.06	0.06	0.06	0.07	0.06	0.05	0.06	0.06	0.07	0.08	0.06	0.05	0.07	0.06	0.07	0.08	0.06
Sugar and confectionery	0.94	0.85	1.16	1.11	1.19	1.38	1.06	0.96	1.31	1.26	1.34	1.60	1.14	1.03	1.39	1.32	1.43	1.65
Drinks	0.57	0.41	0.58	0.31	0.37	0.40	0.53	0.38	0.51	0.27	0.36	0.38	0.73	0.52	0.63	0.38	0.47	0.52
Herbs, spices & condiments	0.04	0.21	0.16	0.06	0.18	0.11	0.04	0.20	0.16	0.05	0.18	0.10	0.04	0.21	0.16	0.06	0.18	0.11

NBW, normal body weight ; OVW, overweight; OB, obese

Table S5 Clinical features of patients from MEDIGENE collection stratified by gender

**Parameter**	**Female**	**Male**	**P-value**
n	73	46	NA
Age (years)	46.50 ± 1.17	48.28 ± 1.21	NS
BMI (kg/m^2)	28.21 ± 0.68	27.82 ± 0.47	NS
Obesity (%)	41.09	26.08	NS
Glycemia (mmol/L)	5.37 ± 0.15	5.92 ± 0.31	NS
Fast insulin (mU/ml)	8.22 ± 0.61	7.06 ± 0.69	NS
HOMA-IR	2.04 ± 0.22	1.76 ± 0.21	NS
Insulin resistance (%)	31.50	23.91	NS
TG (mmol/L)	115.25 ± 7.26	134.16 ± 9.9	NS
HDL (mmol/L)	55.74 ± 2.01	48.25 ± 1.77	NS
MetS-ATPIII (%)	38.03	52.27	NS
Energy intake kcal/d	1969.13 ± 54.29	2252.15 ± 94.1	0.006
Protein intake (g/d)	73.97 ± 2.24	81.22 ± 3.70	NS
Leucine (g/d)	5.76 ± 0.18	6.16 ± 0.28	NS
Isoleucine (g/d)	3.39 ± 0.11	3.63 ± 0.17	NS
Valine (g/d)	3.76 ± 0.11	4.04 ± 0.18	NS
Total BCAA (g/d)	12.91 ± 0.41	13.84 ± 0.63	NS

Table S6. Mean intake of total and individual BCAA (g/d) stratified by BMI* in MEDIGENE surveys

**Mean intake (g/d)**	**Females**				**Males**			
	Normal weight (n =43)	Obese (n = 30)	P-value§	Energy-adjusted P-value	Normal weight (n = 34)	Obese (n =12)	P-value§	Energy-adjusted P-value
Isoleucine	3.17 ± 0.14	3.69 ± 0.19	< 0.0328	0.2327	3.66 ± 0.18	3.53 ± 0.37	0.7297	< 0.2998
Leucine	5.35 ± 0.22	6.35 ± 0.29	< 0.0071	0.0961	6.13 ± 0.31	6.27 ± 0.66	0.8346	< 0.276
Valine	3.57 ± 0.14	4.03 ± 0.17	< 0.052	0.1354	4.08 ± 0.19	3.94 ± 0.40	0.7353	< 0.2024
BCAA	12.10 ± 0.51	14.05 ± 0.63	< 0.0191	0.1387	13.87 ± 0.69	13. 88 ± 1.47	0.9989	< 0.2399

*Obesity was considered with BMI > 30 kg/m^2

§ANOVA test; Values are expressed as mean ± SE

Table S7. Mean intake of total and individual BCAA (g/d) stratified by gender in MEDIGENE surveys compared to other international studies.

	**MEDIGENE***		**Ishikawa-Takata & Takimoto [61]**		**Suga et al. [40]**		**Suga et al. [63]**		**Ishihara et al. Cohort I [62]**		**Ishihara et al. Cohort II [62]**	
Mean intake (g/d)	F (n = 73)	M (n = 46)	F	M	F	M	F	M	F	M	F	M
Isoleucine	3.39	3.63	3.70	4.40	2.85	3.37	2.84	3.40	3.51	4.22	3.421	4.10
Leucine	5.76	6.16	6.50	7.60	5.09	6.03	5.06	6.08	6.07	7.31	5.912	7.09
Valine	3.76	4.04	4.50	5.20	3.36	3.98	3.34	4.01	4.20	5.05	4.11	4.91
BCAA	12.91	13.84	14.70	17.20	11.31	13.38	11.24	13.49	13.78	16.59	13.44	16.10
